# Supplementary material for: Deactivation of redox mediators in lithium-oxygen batteries by singlet oxygen
Source: Nat Commun. 2019 Mar 26;10:1380. doi: 10.1038/s41467-019-09399-0 (PMC6435713; doi:10.1038/s41467-019-09399-0)
Supplement: Supplementary file 1 — Supplementary Information [file 41467_2019_9399_MOESM1_ESM.docx]

**Supplementary Information**

**Deactivation of redox mediators in lithium-oxygen batteries by singlet oxygen**

Kwak *et al*

**Supplementary Figures**


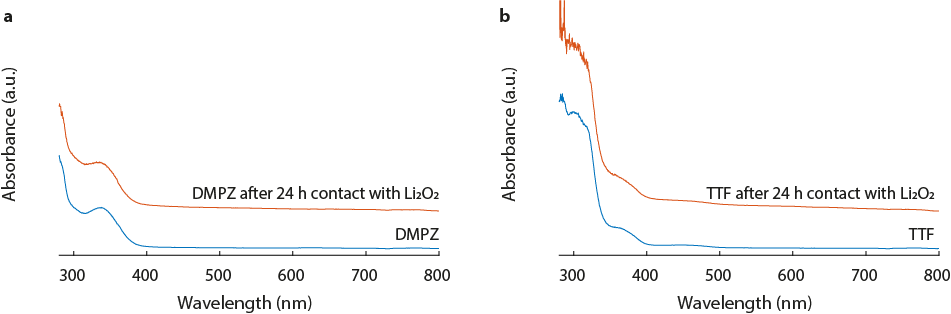


Supplementary Figure 1. Stability characterizations. Stability of DMPZ (a) and TTF (b) in contact with Li_2_O_2_. 60 µM DMPZ or 60 µM TTF solutions, respectively, in 0.1 M LiTFSI/TEGDME were stirred for 24 h with ~1 mg Li_2_O_2_. Spectra were taken before and after contact with Li_2_O_2_ and are offset for better visibility.


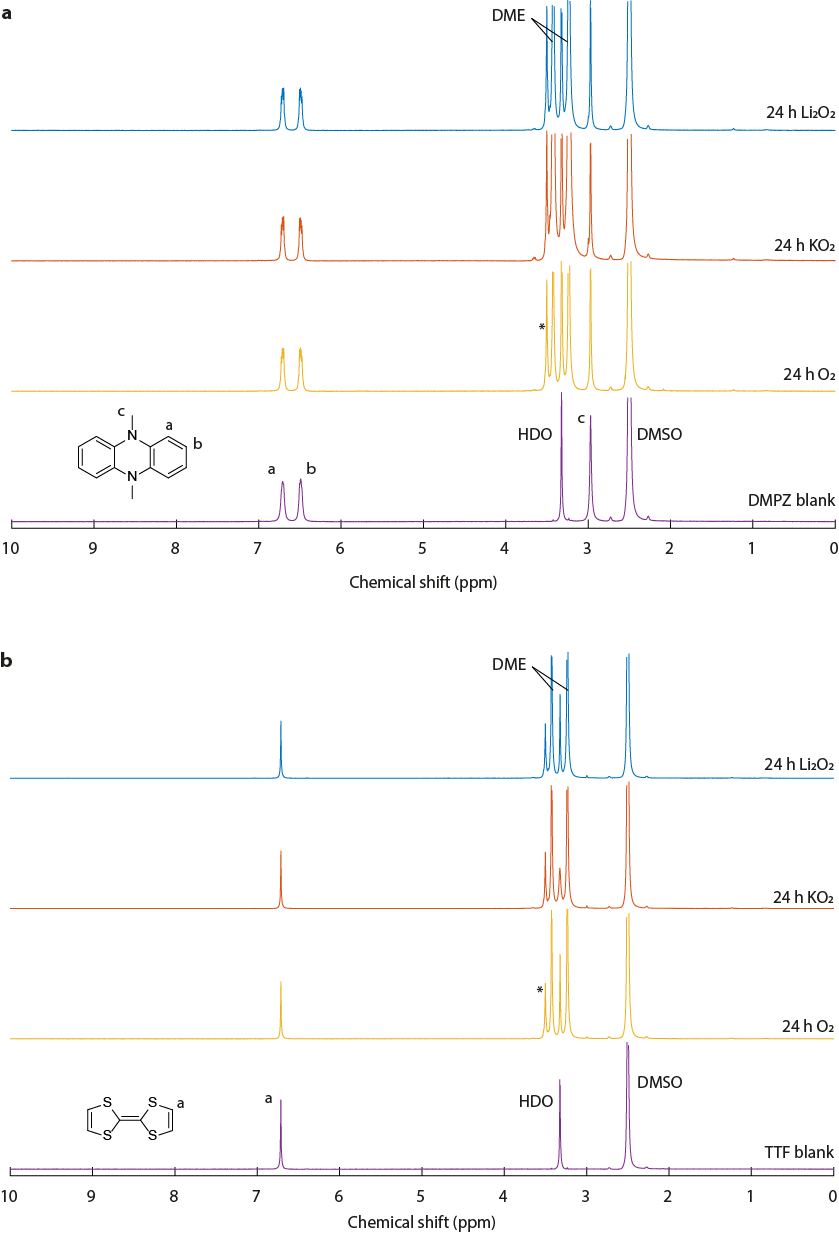


Supplementary Figure 2. Stability of DMPZ and TTF in contact with O_2_, KO_2_ and Li_2_O_2_. ^1^H-NMR spectra (in DMSO-d_6_) were recorded before and after 24 h of contact with the compounds. 1 mg DMPZ or TTF in DME were stirred under O_2_ headspace, or stirred with ~1 mg KO_2_ or Li_2_O_2_. The DMSO peak is taken as internal reference for quantitative comparison of spectra. The * denotes a residue from DME evaporation, which amounts to a content of ~1 ppm in the DME.


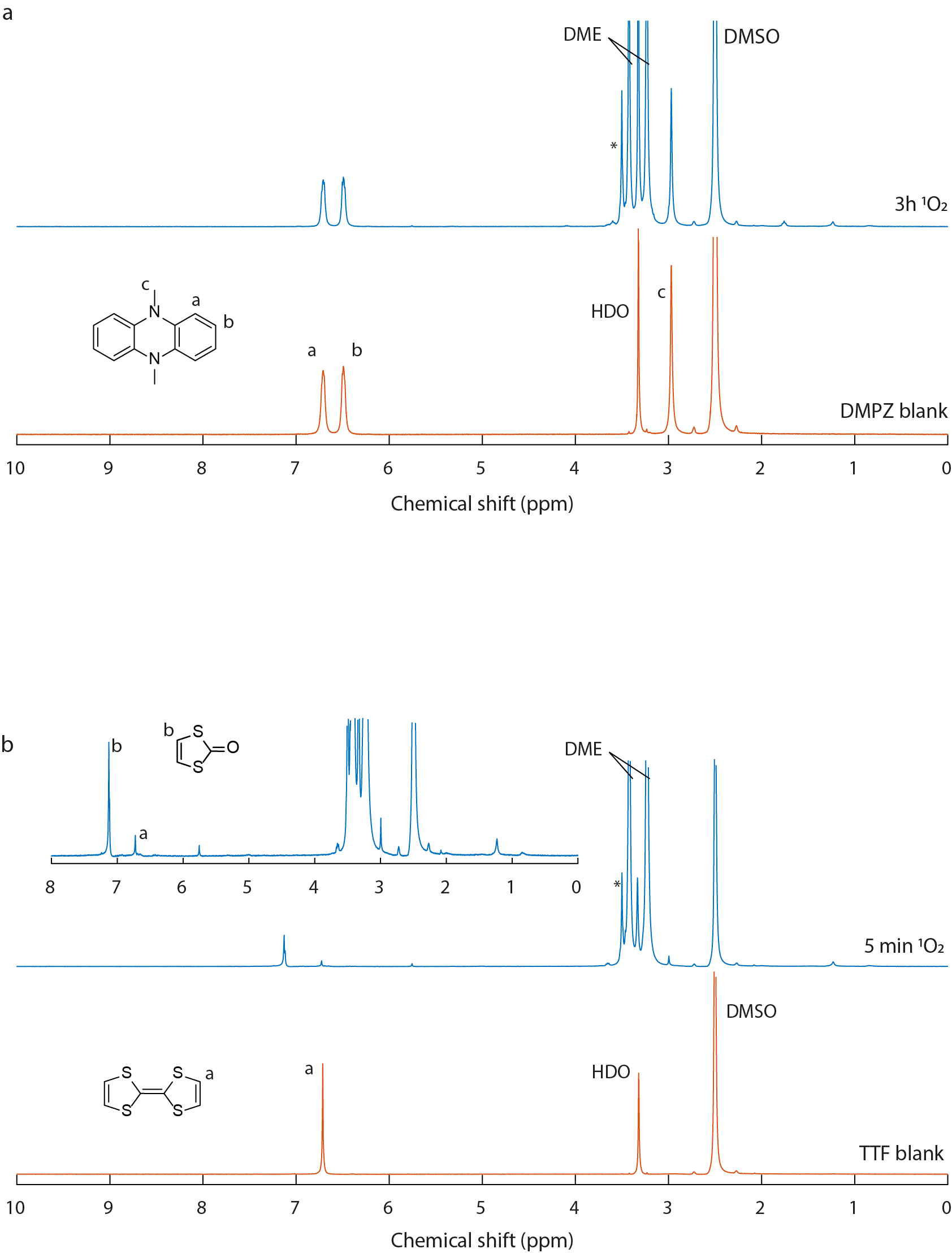


Supplementary Figure 3. Stability of DMPZ and TTF in contact with ^1^O_2_. ^1^H-NMR spectra (in DMSO-d_6_) were recorded before and after photooxygenation. 1 mg DMPZ or TTF in DME was stirred under an O_2_ headspace. For photochemical ^1^O_2_ generation, 1 µM of the photosensitizer palladium(II) *meso*-tetra(4-fluorophenyl)-tetrabenzoporphyrin (Pd_4_F) was dissolved in the solution and illuminated with 643 nm radiation. The DMSO peak is taken as internal reference for quantitative comparison of spectra. The * denotes a residue from DME evaporation, which amounts to a content of ~1 ppm in the DME.

Supplementary Figure 4. Decomposition of TTF to 1,3-dithiol-2-one according to the mechanism proposed in the literature^1^.


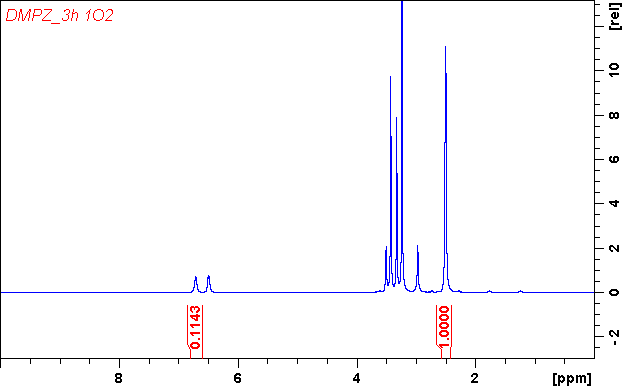


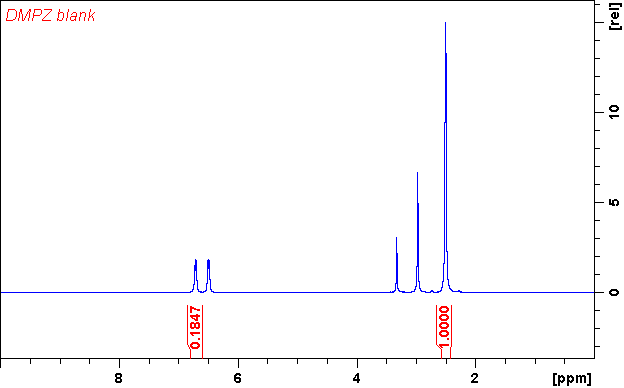


Supplementary Figure 5. Integrals of the ^1^H-NMR spectra in Supplementary Fig 3a.

Supplementary Figure 6. Charge curves for the oxidation of the RMs to RM^+^. 50 μL electrolyte (0.02 M RM (DMPZ or TTF) and 0.1 M LiTFSI in TEGDME) were charged at 100 µA in a Swagelok-type cell using a Li_1−_*_x_*FePO_4_ counter electrode and a carbon paper working electrode with a diameter of 10 mm.


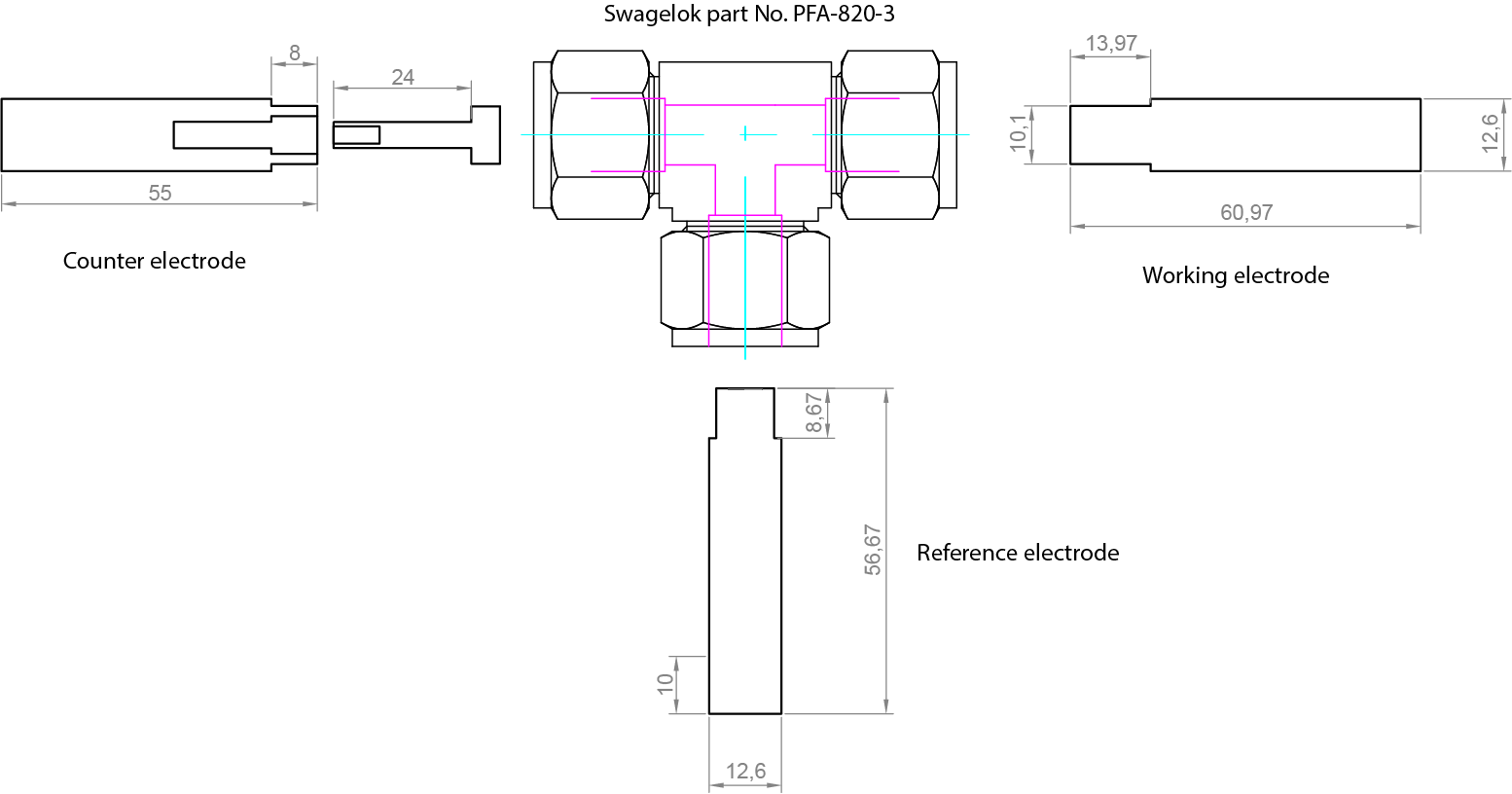


Supplementary Figure 7. The cell design used for electrochemically oxidizing the RM to RM^+^. The central piece is a union tee tube fitting from the company Swagelok (<https://www.swagelok.com/en/catalog/Product/Detail?part=PFA-820-3>) made from perfluoroalkoxy polymer (PFA). The plungers for counter, working and reference electrodes are made from stainless steel (grade SAE 316L).

**Supplementary Reference**

1. Zhang, X., Lin, F. & Foote, C. S. *J. Org. Chem.* **60**, 1333 (1995)
